# Sn-Based Perovskite Solar Cells towards High Stability and Performance

**DOI:** 10.3390/mi14040806

**Published:** 2023-03-31

**Authors:** Wafa’ Ayaydah, Eman Raddad, Zafer Hawash

**Affiliations:** Department of Physics, Birzeit University, Birzeit, Ramallah 71939, Palestine

**Keywords:** tin perovskites, stability, power conversion efficiency, crystallization, charge recombination

## Abstract

Recent years have witnessed rapid development in the field of tin-based perovskite solar cells (TPSCs) due to their environmental friendliness and tremendous potential in the photovoltaic field. Most of the high-performance PSCs are based on lead as the light-absorber material. However, the toxicity of lead and the commercialization raise concerns about potential health and environmental hazards. TPSCs can maintain all the optoelectronic properties of lead PSCs, as well as feature a favorable smaller bandgap. However, TPSCs tend to undergo rapid oxidation, crystallization, and charge recombination, which make it difficult to unlock the full potential of such perovskites. Here, we shed light on the most critical features and mechanisms affecting the growth, oxidation, crystallization, morphology, energy levels, stability, and performance of TPSCs. We also investigate the recent strategies, such as interfaces and bulk additives, built-in electric field, and alternative charge transport materials that are used to enhance the performance of the TPSCs. More importantly, we have summarized most of the recent best-performing lead-free and lead-mixed TPSCs. This review aims to help future research in TPSCs to produce highly stable and efficient solar cells.

## 1. Introduction

Nowadays, perovskites solar cells (PSCs) are considered as one of the most promising clean and renewable energy technologies. PSCs have achieved a high power conversion energy (PCE) up to about 26% [1,2], making it one of the most compelling research fields. Additionally, PSCs have enormous potential and versatile applications starting from solar plants for daily use electricity up to being utilized in space applications due to their appealing properties (light weight, low cost, flexibility, high PCE, and radiation tolerance) [3]. The ideal crystal structure of perovskites is cubic and based on the formula ABX_3_. The cation ‘A’ (organic or inorganic such as methylammonium (MA), formamidinium (FA), or Cs) occupies the vertices of the cubic lattice. The ‘X’ anions (oxygen or halogen (Cl^−^, Br^−^, or I^−^)) are located at the faces. The ‘B’ cation (Cu^+2^, Sn^+2^, or Pb^+2^) occupies the octahedral site. Perovskites containing halides were introduced for the first time in 1978 [4]. However, the first PSC with halides was reported in 2009 [5].

Currently, all high-performance PSCs contain Pb in the light absorber since it is proven to provide superior opto-electronic properties, such as high absorption coefficient [6] and excellent transport capability [7]. However, lead toxicity and the correlated need for large-scale commercialization of PSCs raise concerns about the hazards that Pb may cause to humans and the environment [8]. Pb can rapidly enter the human bodies through inhalation, causing dangerous effects on the organs of the body even a on molecular level [9]. Therefore, there is an urgent need to replace the toxic lead with elements that have less toxicity or none. Some of the suggested alternatives are Sn, Sb, Bi, or Ge [10,11,12,13,14,15]. Between these fundamental elements, Sn presents itself as one of the most promising options [16,17,18,19,20,21,22]. Sn is a group 14 element in the periodic table, and it has a similar ionic radius (115 pm) to Pb (119 pm), allowing for ASnX_3_ perovskites to develop [19]. In comparison to the Pb-based perovskites, Sn-based perovskites exhibit similar superior optoelectronic properties, with a narrower bandgap of about 1.3 eV [23], high charge mobilities of about 600 cm^2^.V^−1^s^−1^ [24], long carrier diffusion and lifetime [25], and high absorption coefficients (>10^−4^ cm^−1^) [26].

Based on the detailed balance limit, the evaluated value of the PCE of the Sn-based perovskite solar cells (TPSCs) is about 30% [27,28]. However, the PCE that is reported until now is much lower than that for Pb-based perovskites. The poor stability of Sn perovskites in a moist environment [29], as well as the presence of oxygen [30], under light irradiation [31], applied electric field [32], thermal stresses [33], and defects in the perovskite materials [34], all contribute to the often observed low PCE. Tin (i.e., Sn^+2^) oxidizes fast to form Sn^+4^ due to its tendency to be more stable. It functions as a p-type dopant in the structure, resulting in an excessively high dark-carrier concentration and extremely high photo-carrier recombination [35,36,37]. It has low formation energies, which results in the creation of Sn vacancies, causing large amounts of self-doping in perovskite films and resulting in further non-radiative recombination losses [38]. Therefore, the performance and progress of the TPSCs is still behind the Pb-based PSCs and exhibiting significantly lower bioavailability.

In recent years, enormous progress and research on TPSCs have been reported and conducted. This review summarizes the most important aspects as well as the recent progress of TPSCs. The basic and most common device structure and the characteristics of each layer of TPCS are discussed and compared with those of Pb-based PCSs. The factors that are affecting the performance and stability of TPSCs, including additives (to precursor or at the interface), crystallization, strains, morphology, built-in electric field, charge extraction, energy levels, alternative charge transport materials, and solvents are presented and discussed.

## 2. Sn Perovskite Materials

### 2.1. High Conductivity and Metallic Behavior

**Figure 1:**
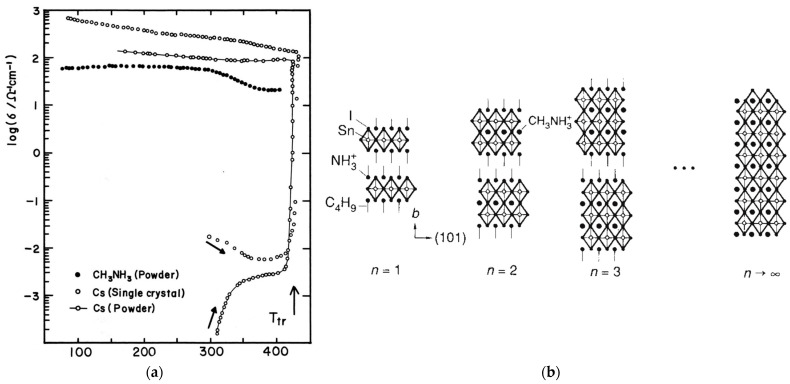
(**a**) Electrical conductivity as a function of temperature of CsSnI_3_ and MASnI_3_. Reproduced with permission [39]. Copyright 1990, De Gruyter, and (**b**) schematic representation of BA incorporation in perovskite materials with n = 2, 3, and ∞, reproduced with permission [43]. Copyright 1995, Nature publishing group.

A very important insight about the electronic properties of Sn-based perovskites goes back to the work reported by Yamada et al. [39] in 1990. They investigated a set of different ASnI_3_ perovskites (A = K, NH_4_, Rb, Cs, or MA). Both of the investigated Cs and MA -SnI_3_ perovskites exhibited high conductivity at room temperature (Figure 1a), which was found to be in the range of 10^2^–10^3^ S cm^−1^ [39]. However, in the case of CsSnI_3,_ this conductivity that is similar to metallic materials was only observed after heating, where the films were observed to undergo structural transformation at 425 K. This structural change was not observed in the case of MASnI_3_. The heating treatment increased the conductivity of CsSnI_3_ by about 4 orders of magnitude and at the same time the materials’ color was observed to change from greenish to black with a metallic luster. Additionally, the conductivity of the Cs-based sample was observed to increase with decreasing temperature in a similar trend, which is usually observed for metals [39].

In 2013, Stoumpos et al. [40] calculated the electronic properties of the of different Sn perovskites using the Seebeck coefficient and Hall effect measurements. An exceptionally high electron mobility of MASnI_3_ was calculated, up to 2320 cm^2^ V^−1^·s^−1^. In 2016, Ma et al., reported that MASnI_3_ may have opto-electronic properties that are better than those of CH_3_NH_3_PbI_3_ [26]. They reported long carrier diffusion lengths of about 300 nm for electrons and 200 nm for holes (and can increase 10 times when the SnF_2_ reducing agent was used), which were recorded using time-resolved fluorescence spectroscopy [26]. On the other hand, an interesting slow relaxation of the hot carriers (0.5 ps) was observed for the same samples [26]. Fang et al. [41] also found that the hot carriers in FASnPbI_3_ have lifetimes of a few seconds, which can be utilized to design solar cells with PCEs that can exceed the Shockley–Queisser limit [27]. Additionally, with increased excitation source power, they observed a 75 meV blue shift of the optical bandgap at room temperature, which is due to the filling of band edge states [41]. However, the electronic properties are very dependent on the preparation method, which may directly affect the material quality and stoichiometry [40,42].

### 2.2. Metallic to Semiconducting by Incorporation of Different Organic Cations

In 1994, looking for new classes of superconductors, the insertion of organic chains of n-butylammonium (BA) into MASnI_3_ was performed and investigated by Mitzi et al. [43]. The incorporation of BA in the MASnI_3_ yielded a semiconducting behavior. With a general formula, BA_2_MA_(n−1)_ Sn_(n)_I_(3n+1)_. When n = ∞, the film only consists of MASnI_3_ and was observed to be also more of metallic. With n = 2, the formula becomes BAMASnI_3_ and the measured resistivity was observed to be 10 Ω cm at room temperature. With more BA incorporated (smaller n values), a more nonmetallic behavior was observed, (i.e., n = 3) along with a trend of resistivity decrease up to a temperature of 75 K followed by a slow upturn in resistivity with higher temperatures.

Similarly, an FA cation was also incorporated in the same way as BA [44]. Based on the formula [NH_2_C(I)=NH_2_]_2_(CH_3_NH_3_)_m_ Sn_m_ I_3m+2_, with higher m values a metallic behavior was observed (Figure 1b). When n, m ⇒ ∞, the material is just MASnI_3_ [43,44]. Mitzi et al. [45] further investigated the observed metallic behavior of MASnI_3_. They also observed a low carrier density (p-type metallic behavior) with a hall carrier density of 2 × 10^19^ cm^−3^. The resistivity was also observed to decrease with decreased temperature, which is in line with the metallic behavior, with a resistivity at room temperature of about 7 mΩ cm [45]. Moreover, Mitzi et al. [45] also reported that the prepared MASnI_3_ material has free carrier IR (infra-red) reflectivity with a plasma edge, which confirms the metallic nature of the prepared materials. 

After that, in 1997, the same previous group reported that the resistivity of MASnI_3_ at room temperature in the pure FASnI_3_ is about three orders of magnitude higher than previously reported [46]. The resistivity at room temperature of FASnI_3_ was found to be ~7 Ω cm compared to 3 Ω cm in the case of MASnI_3_. The use of FA resulted in lattice expansion, which was speculated to have an effect on the formation of defects, which would readily produce a shift in the energy levels and affect transport properties [46]. Additionally, FA has an extra NH_2_ group compared to MA, which provides an extra site to form an additional hydrogen bond that can contribute to better ordering. In general, the FAMA system was also observed to add additional freedom to tailor the electronic properties of such materials by changing the ratio from MA to FA [46].

### 2.3. Importance of Preparation Method

Several comparative studies of CsSnI_3_’s electrical behavior and a detailed investigation of MA and FA compared to Cs-based -SnI_3_ perovskites were reported [24,42,47]. However, the preparation method and post-treatment can majorly alter the film electronic nature up to even an n-type semiconductor [40]. Two of three methods used to prepare Cs, FA, and MA-SnI_3_ perovskites (single crystals from solution, and compressed pellets from sealed tubes dry reaction) exhibited high resistivity trends that are consistent with semiconductor behavior [40]. However, similar samples prepared using the third method (dry in an open tube) were found to behave as a p-type metal. The single crystals from the solution method of FA and MA-SnI_3_ were found to be n-type semiconductors with a low level of electron carriers in the temperature range from 300 to 400 K. However, pressed pellets from the dry method with no annealing were found to be p-type semiconductors. When high-quality ingots of CsSnI_3_ were prepared, superior performance with lifetimes approaching 6.6 ns and diffusion length approaching 1µm compared to only 54 ps and 16 nm for the lower quality polycrystalline ones were observed [25]. For these ingots, no reducing agents such as SnF_2_ were used. Based on the extracted lifetimes and diffusion lengths of these high-quality CsSnI_3_ crystals, a PCE of 23% was estimated [25]. It was shown that the significant factor for electrical properties of the Sn perovskites is the easy oxidization of Sn^+2^ and the formation of the more stable Sn^+4^. Consequently, p-type metal character and low resistivity are increased, contributing to the self-doping mechanism. Sn has two active electrons on the 5-S orbital, which causes the easy oxidization from Sn^+2^ to Sn^+4^. It tends to lose these two electrons because the fully occupied 4d orbital electrons are less effective as electromagnetic shields [48].

### 2.4. Defect Physics of Sn Perovskites

**Figure 2:**
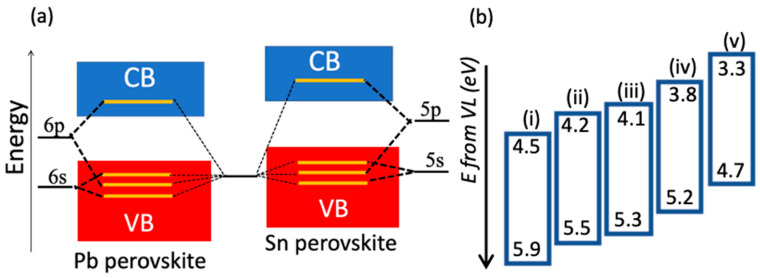
Energy of Sn perovskite bands compared to Pb perovskite and often observed discrepancy of measured and reported values of Sn perovskites’ properties, manifested in the bandgap, and the position of valence and conduction bands (VB and CB, respectively) with respect to vacuum level (VL). (**a**) Higher energy band of Sn-perovskites, and (**b**) Discrepancy of energy reported values of FASnI_3_ (**i**) [53], (**ii**) [54], (**iii**) [55], (**iv**) [56], and (**v**) [57].

Tin-based perovskites have a direct bandgap, meaning that the momentum of the VB maximum and the CB minimum is the same [10]. This results in a high absorption coefficient. However, it is reported to be an order of magnitude less than Pb-based perovskites [10,26,49]. Even though Sn perovskites have a direct bandgap, their energy bands are higher than those of Pb-based perovskites (Figure 2a). As a result, the Sn-I bond can be broken easily, allowing for I^−^ and Sn^+2^ to react with H_2_O and O_2_, respectively; therefore, the Sn perovskites may easily become poor in crystal quality and photovoltaic performance and the properties may be easily altered.

In Sn^+2^ states, the 5P orbital’s high energy causes Lewis acidity [50], which leads to the unwanted and uncontrolled rapid crystallization of perovskites. This leads to a substantial amount of physical defects in crystal lattice, which may be the reason for the often observed discrepancy of Sn perovskite properties, such as the energy levels measured and reported values of Sn perovskite films (Figure 2b). LaMer’s classic crystal growth theory states that small crystal grains could form due to fast crystallization [51]. These grains significantly affect the optoelectronic properties of Sn-based perovskites since it helps to ease the losses of Sn ions [52]. On the other hand, the oxidization from Sn^+2^ to Sn^+4^ leads to the formation of Sn vacancies (V_Sn_) and act as high p-doping. Finally, these defects result in hole carriers with high concentrations, which operate as recombination centers for charge carriers. Consequently, tin-based perovskite systems perform sub-optimally.

## 3. Stability of Sn Perovskites

The low environmental stability of Sn perovskites has been long recognized as a significant issue, and it has been blamed for their inability to achieve performance comparable to Pb-based PSCs. Perovskite layers tend to oxidize rapidly with oxygen in ambient conditions [58,59]. As a result, short carrier lifetimes and a high nonradiative carrier recombination rate are often observed and detrimental to their photovoltaic performance. There are several factors leading to the well-known poor stability of the Sn-based perovskites, including extrinsic and intrinsic factors. We will discuss specifically the following factors: oxygen, moisture, illumination, and ion migration.

### 3.1. Moisture Effect

Moisture is considered to be the most prominent factor that causes the degradation of the Sn-based perovskites, leading to the oxidation of Sn [60]. In the presence of moisture, the perovskites are expected to degrade to form metal iodides (i.e., SnI_2_). Simultaneously, in the case of MA cations (CH_3_NH_3_I), they form HI acid under H_2_O environments. Even though minimal moisture is necessary to decompose the organic part, an excess is needed to break down the HI and CH_3_NH_2_ derivatives for the degradation to progress.

**Figure 3:**
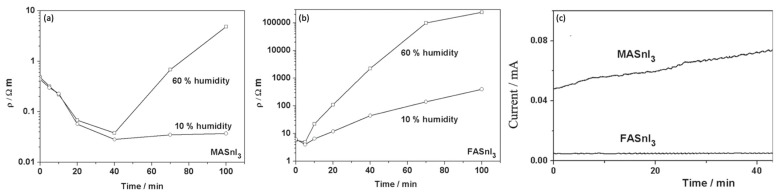
As a function of time and humidity, the resistivity of (**a**) MASnI_3_ and (**b**) FASnI_3_. (**c**) In a glovebox in the dark, the MASnI_3_ and FASnI_3_ conductor structures were characterized by the current they carry when biased by 1 V. Reproduced with permission [63]. Copyright 2016, WILEY-VCH.

In addition to MA cations, FA cations also have a hygroscopic nature [61,62]. FA and MA cations also react with H_2_O in distinct binding mechanisms [63]. FA and H_2_O form stronger hydrogen bonds (than MA^+^), increasing the size of the perovskite lattice and reducing the stability of the crystal structure. However, this may reduce the H_2_O interaction with SnI_2_. Furthermore, the changes that happen to the electronic properties of FASnI_3_ and MASnI_3_ in the presence of moisture air include the fact that the resistivity decreases at the beginning of air exposure for both films and then increases over time [63]. After 40 min of air exposure with 60% humidity, the MA perovskite displays a drop in order of magnitude. On the other hand, the FA perovskite displays a 10% drop under similar conditions (Figure 3a,b). 

When TPSCs are tested in nitrogen-filled gloveboxes with H_2_O ~ 1 ppm, the performance of FASnI_3_ devices is more stable and they display good reproducibility (Figure 3c). Therefore, it may be possible to produce better TPSCs by carefully selecting the organic cation, which reduces moisture ingress [64].

### 3.2. Oxygen Effect

**Figure 4:**
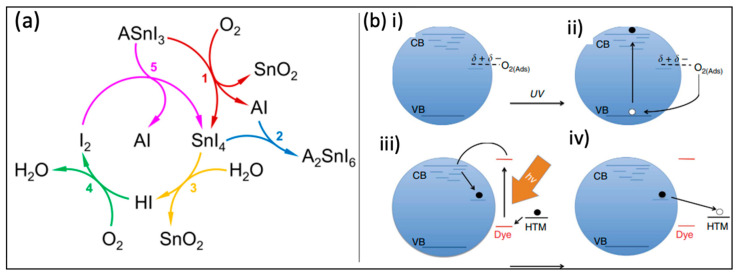
(**a**) Cyclic degradation of tin iodide perovskite in the presence of humid air in which reaction 1 is the oxidation of ASnI_3_ By O_2_, reaction 2 is the solid-state formation of A_2_SnI_6_, reaction 3 is the hydrolysis of SnI_4_ by H_2_O, reaction 4 is the oxidation of HI By O_2_, and reaction 5 is the oxidation of perovskite by I_2_. Reproduced under terms of CC-BY [65]. Copyright 2021, Nature publishing group. (**b**) Proposed ultraviolet light degradation process when photo-generated holes are applied externally with oxygen, after which they react with oxygen radicals adsorbed on the surface of oxygen vacancies, as seen in (**i**,**ii**). As a result of the oxygen, deep surface traps remain unfilled, and each trap has a free electron. (**iii**) As a result of the sensitizer’s photoexcitation, electrons flow into the conduction band, where they are deeply trapped, or directly into the deep surface traps, and as a result of that the electrons are strongly trapped and they easily recombine with holes in the spiro-OMeTAD (2,2’,7,7’-Tetrakis[N,N-di(4-methoxyphenyl)amino]-9,9’-spirobifluorene) hole transporter layer (**iv**). Reproduced with permission [67]. Copyright 2013, Nature publishing group.

Oxygen damages the charge neutrality of the perovskite structure and causes it to disintegrate into oxides or hydroxides of Sn and MAI or FAI, and as a result the electron–hole recombination increases [10]. The organic cations (i.e., FA and MA) have a significant impact on the O_2_-induced degradation of Sn-based perovskites, as well as affecting the optical and electronic properties of the materials such as charge mobility, band alignment, and diffusion length [63]. Lanzetta et al. [65] defined the mechanisms underlying the degradation of different dimensional tin–perovskite films based on phenethylamine (PEA)_0.2_(FA)_0.8_SnI_3_. As a result of moisture and oxygen, SnI_4_ and SnO_2_ were formed and resulted in the formation of molecular iodine, which is detrimental to the perovskites’ performance. Iodine is a hyper aggressive compound that can further oxidize the perovskite, resulting in a cyclic degradation (Figure 4a) [65,66].

### 3.3. Illumination

In general, TiO_2_ is one of the most common electron transport materials (ETMs) for n-i-p PSCs. Specifically, under UV-illumination, TiO_2_ molecules are a typical photo-catalyst that can accelerate the degradation of perovskite materials [67]. TiO_2_ has many several oxygen vacancies as it is an n-type material. In the presence of oxygen, it adsorbs these vacancy cites due to the reaction with the deep electrons that lie in the vacancies. Through UV-illumination, an electron–hole pair was formed in TiO_2_. As a result, the electron in the vacancy, which lies before the CB by ~1 eV, will interact with holes in the VB. This operation leads to free electrons in the CB and empty oxygen vacancies with a positive charge, which will act as traps for electrons generated from perovskite material. Additionally, in the common n-i-p structure, spiro-OMeTAD hole transport material (HTM) is used as a heavily p-doped material that will allow for recombination with these free electrons in the CB (Figure 4b(ii–iv)). Lee et al. [68] mitigated this problem in PSCs replacing mesoporous TiO_2_ with a thin layer of insulating Al_2_O_3_. However, with this UV-related problem and with the aforementioned TiO_2_-related problems, the p-i-n structure is more commonly used for TPSCs.

One of the popular methods to improve the illumination stability of Sn perovskites is with ‘X’ site (ABX_3_ perovskites) doping, which is unfortunately more susceptible to illumination degradation due to phase separation under illumination [69]. Additionally, the ‘A’ organic cations, especially MA^+^ (best for highest PCEs), are very prone to decomposition under illumination [70,71]. Therefore, ‘A’ site manipulation or additives are the most popular methods to enhance the Sn perovskites’ stability under illumination [72]. 

### 3.4. Ion Migration

Ion migration is a well-known and an undesirable phenomenon in PSCs. This is also related to the hysteresis in current density-voltage (*J-V*) characteristics curve [73]. Due to the dissociation of the charged ions, the diffusion of the ions results in unbalanced charge transport [74]. It also has an impact on the cell’s long-term stability and performance, as well as its photoelectronic properties. Defects/ions such as iodide vacancies can migrate across the contact during PSC operation, producing interfacial deterioration, compromising device function, and even triggering device failure. The activation barrier of hybrid perovskites is low. Thus, the ions, such as iodide, are induced to migrate through perovskite devices under applied bias or irradiation [73]. The ions of the iodide (I^−^) easily move to defects in perovskites under an external electric field [75]. Additionally, excess iodide may accumulate at the interface between the perovskite, charge transport materials, and electrodes. As a result, the solar cells perform poorly and degrade rapidly [76].

One common method to restrict the ion migration is using additives. For example, the addition of polyvinyl alcohol (PVA) polymer to the FASnI_3_ solution was shown to improve hydrogen-bonding interactions. Therefore, the PVA-inhibited transport of iodide ions prevented the formation of iodide vacancies, and lowered ionic conductivity, preventing ion migration and preserving the long-term stability of tin-based PSCs [77]. More examples of the recent development of enhanced stability are presented in a later section.

## 4. Basic Structure and Charge Transport Materials for Tin-Based Perovskite Solar Cells

A typical PSC consists of five main individual layers deposited consecutively on a glass or flexible substrate. Based on the order of the deposited layers on the transparent conductive electrode (TCE), it can be a normal or an inverted structure. The normal structure is TCE/ETM/perovskite absorber (PVSK)/HTM/top electrode (n-i-p stack). The inverted structure is TCE/HTM/PVSK/ETM/top electrode (p-i-n stack). 

**Figure 5:**
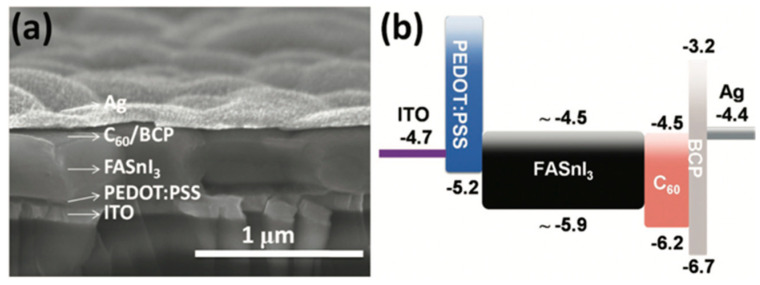
(**a**) SEM image of an inverted FASnI3 perovskite solar cell with SnF2 additive at 10 mol%. (**b**) System energy level diagram of FASnI3. Reproduced with permission [53]. Copyright 2016, WILEY-VCH.

One of the first TPSCs with the n-i-p structure was reported by Hao et al. [11] with a standard architecture of fluorinated tin oxide (FTO)/compact TiO_2_/MASnI_3_/spiro-OMeTAD/Au and achieved a PCE of 5.7%. The first TPSC with a p-i-n structure was reported with indium tin oxide (ITO)/poly(3,4-ethylenedioxythiophene):polystyrene sulfonate (PEDOT:PSS)/FASnI_3_/fullerene (C_60_)/2,9-dimethyl-4,7-diphenyl-1,10-phenanthroline (BCP)/Ag with PCE of 6.22% (Figure 5) [53]. These structures had low efficiencies with many problems and challenges, such as poor and uncontrolled quality of Sn perovskites as well as poor interfacial contact resulting in instability problems, mismatch of energy levels, insufficient charge extraction, and hysteresis effect. However, inverted (p-i-n) TPCSs exhibited a slightly better performance, stability, lower cost, as well as ease of fabrication, which made them more reliable to fabricate.

### 4.1. Electron Transport Layer (ETL)

The ETL is necessary to transport photo-generated electrons from the perovskite layer towards the anode. One of the most common ETLs is the compact blocking layer of TiO_2_, which is usually deposited using spray pyrolysis and it has a good chemical stability, electrical properties, and is non-toxic [78]. However, TiO_2_ implies high-temperature processing, low durability due to brittle nature, and is a photocatalyst that tends to adsorb oxygen, which results in the degradation of perovskites upon UV intake. Due to this problem, researchers attempt to find alternatives for TiO_2_. One of the possible alternatives is ZnO, which exhibits a suitable bandgap, similar to TiO_2_ (3.2 eV). It also has a high electron mobility equal to ~200 cm^2^ V^−1^ s^−1^ [79]. However, ZnO has a poor stability and is hygroscopic, which makes it less suitable due to its degradation and fast reaction with air [80]. Another rising alternative is SnO_2_, which has a high electron mobility (250 cm^2^ V^−1^ s^−1^, which is higher than that for TiO_2_ and ZnO) [79]. Its bandgap is wider (3.8 eV), which is more suitable for matching with the perovskite materials’ bandgap [80,81]. In addition, it is less hygroscopic, which results in lower photocatalytic activity and therefore stable performance [82,83,84]. An example from 2018, SnO_2_ with a thin layer of C_60_ as ETM in n-i-p TPSCs with the architecture of ITO/SnO_2_/C_60_/FASnI_3_/spiro-OMeTAD/Ag was able to achieve 4.34% without noticeable problems [85]. 

The most common ETM material used in inverted devices is C_60_ and [6,6]-phenyl-C61-butyric acid methyl ester (PCBM). Fullerenes show a good band alignment with perovskite materials, leading to lower charge recombination since it transfers smoothly to the perovskite [86,87]. It also has a good energy alignment with the CB of the FASnI_3_ perovskites. Furthermore, using C_60_ as ETM exhibits low hysterias in the *J-V* curve, leading to a high PCE [88]. 

### 4.2. Hole Transport Layer (HTL)

The most common hole transport layers (HTLs) that are used in high-performance PSCs are spiro–OMeTAD [89], poly[bis(4-phenyl)(2,4,6-trimethylphenyl)amine] (PTAA) [90,91], PEDOT:PSS [90,92,93], and some metal oxides as well as small molecules [90,94]. HTM plays a significant role in the hole transport and stability of the perovskite layer [95]; however, pure spiro-OMeTAD exhibits low conductivity [96]. Different small molecules are often added as p-dopants, such as lithium salt Lithium bis(trifluoromethanesulfonyl)imide (Li-TFSI) and 4-tert-Butylpyridine (tBP) [97]. Adding these molecules improves the hole mobility, and as a result, high open-circuit voltage (*V_OC_*) and PCE were achieved [98,99]. However, due to high moisture sensitivity of Li-TFSI and tBP, the combination results in degradation; additionally, tBP can dissolve perovskites [89]. In order to solve these issues, researchers introduced a set of alternatives such as PEDOT:PSS [100], which is more common in inverted devices. However, PEDOT:PSS is sensitive to moisture, leading to stability problems. One of the alternatives investigated by Cao et al. [101] was copper thiocyanate (CuSCN), with which a PCE of 7.34% was achieved. Nickel oxide (NiO) is also a common HTM and has the advantage of more stability than another alternatives. On the other hand, it has a wide bandgap and good energy alignment with the Sn perovskite [102]. 

## 5. Thin Films of Sn Perovskites towards High Efficiency and Stability

Compared to Pb-based PSCs, TPSCs exhibit a much lower PCE, mainly due to the poor film quality, correlated degradation, and detrimental effects. Perovskite films are often fabricated from solutions due to ease of fabrication. In order to create a high-performance tin-based PSC, it is imperative to form dense, compact, well-crystalline perovskite films [103]. Many ways have been proposed to resolve the instabilities of tin-based perovskites. The first step to enhance the stability of the device is to gain a deeper understanding of the degradation mechanisms. Earlier, we briefly pointed out the effects of moisture, oxygen, illumination, ion migration, and chemical reactions which are the most common causes of degradation in perovskite halides. In this section, we go over the most effective ways for increasing the performance and stability of Sn-based perovskite halides that have been reported. 

### 5.1. Tin Perovskites with Additives/Reducing Agents

A defect is often induced by the fast oxidation of Sn^+2^ due to its fast kinetics of nucleation and growth in perovskites; therefore, additives that often reduce the fast oxidation and improve the film morphology (compactness) are required. Sn halides (SnF_2_, SnCl_2_, SnBr_2_, and SnI_2_) and several organic molecules have been shown to prevent oxidation and enhance the performance of TPSCs. Furthermore, Sn halide additives are able to compensate for Sn vacancies in the films, improving the film morphology, reducing the likelihood of vacancy formation, and reducing the background hole density [104].

#### 5.1.1. SnF_2_ Additive

One of the first reports of reducing agents was implemented in 2012 by Chung et al. [24] using CsSnI_3_ Sn perovskites as an HTM by doping with SnF_2_ in dye-sensitized solar cells, which helped in producing a *V_OC_* of 0.42 V and an overall PCE of 0.9%. Following this, in 2014 Kumar et al. [105] found that incorporating SnF_2_ into CsSnI_3_ reduces the formation energy of Sn vacancies, leading to less conductivity in CsSnI_3_, and as a result, the TPSCs gained a high current density (*J_SC_*) of 22 mA cm^−2^. The impacts of SnF_2_ doping in FASnI_3_ with 20% mole were confirmed with X-ray diffraction (XRD) data that indicated a reduced amount of Sn^+4^ [42]. This helped the current density to increase by 10 mA cm^−2^. In 2018, Xiao et al. [106] were able to achieve homogeneous crystal growth and uniform film coverage. They demonstrated that SnF_2_ can reduce Sn vacancy (V_Sn_) concentrations by boosting their formation energy. Following that, Hartmann et al. [107] studied the electronic structure of CsSnBr_3_ and observed that Sn oxidation was inhibited by the addition of 20% mole SnF_2_. In 2016, Ma et al. [26] showed that SnF_2_ had the effect of a distinguishable increase in carriers’ lifetime from 0.7 ns to 6 ns. Additionally, hole diffusion length was estimated to increase substantially, as a result of the addition of SnF_2_, whereas the electron diffusion length remained unchanged. SnF_2_ is commonly used in most tin-based photovoltaic systems for easy optimization of Sn perovskites.

#### 5.1.2. SnCl_2_ Additive

An additional reducing agent commonly used in Sn perovskites is SnCl_2_ [108]. It was used to increase the stability of the Sn-based devices in an HTM free structure. Using X-ray photoemission spectroscopy (XPS) analysis on CsSnI_3_ perovskite samples treated with the addition of 10 mol% of SnCl_2_, they found that SnCl_2_ was present at the perovskites’ surfaces, and that the SnCl_2_ layer could act as a dryer to improve the stability of CsSnI_3_. Interestingly, after 5 months of storage under a nitrogen environment, the PCE was observed to increase, along with the *V_OC_* and FF. This improvement in the performance over time can be explained by the SnCl_2_ doping on the electron-transport layer of the used ETM (i.e., PCBM). In addition, they evaluated different tin halide additives (SnCl_2_, SnBr_2_, SnI_2,_ and SnF_2_) to see how they could affect HTM-free TPSCs (ITO/CsSnI_3_/PC_61_BM/BCP/Al). Among the tested devices, with the SnCl_2_ additive, a PCE of 3.56% was the best, and SnCl_2_ resulted in the highest film stability. Performance improvements may be attributable to the enhancement of PCBM ETM crystallization under light illumination.

#### 5.1.3. Hydrazine Additive

Hydrazine has long been used in chemical synthesis to prevent oxidation (reducing agent). Additionally, hydrazine’s highly volatile nature makes it an easy agent to be introduced as a reducing atmosphere. Song et al. [60] introduced hydrazine vapor atmosphere prior to the spin-coating process of the perovskite precursor. The films were formed in hydrazine atmosphere resulting in reduced defects, oxidation, and therefore better performance. Similarly, Kayesh et al. [109] were able to minimize the concentration of Sn^+4^ by 20% and significantly suppress carrier recombination during the fabrication of FASnI_3_ perovskite films, by incorporating hydrazinium chloride (N_2_H_5_Cl) into a single precursor solvent system. A high PCE with significantly enhanced *V_OC_* and pinhole-free FASnI_3_ perovskite films were achieved. Li et al. [110] reported a solution–deposition method for the fabrication of MASnI_3_ that included hydrazinium iodide (N_2_H_5_I) with SnI_2_ precursor. A mesoporous TPSC with a PCE of 7.13% was achieved.

#### 5.1.4. Acidic Additives

Hypophosphorous acid (HPA). In the synthesis of tin-based perovskites, HPA has long been used as a common reducing agent. In most circumstances, HPA is utilized as an assisting reducing agent in antioxidation when powerful agents such as hydroiodic acid (HI) or SnF_2_ are present, eventually stabilizing the process. Researchers used HPA as a coordinating agent in the CsSnIBr_2_ production process, which allowed them to speed nucleation while restricting Sn^+2^ oxidation. Charge carrier density and Sn vacancy levels were lowered as a result of the HPA integration [111].

2,2,2-trifluoroethylamine hydrochloride (TFEACl). In combination with SnF_2_, 5 mol% of TFEACl was found to improve and enhance FASnI_3_ solar cells [100]. The work function of perovskite films may be adjusted by adding Cl. Therefore, the perovskite films are better aligned with the charge transport layers. In addition, light soaking stability was found to be improved, which all resulted in improved device performance and charge collection.

Gallic acid (GA).Wang et al. [112] used the antioxidant GA as an additive together with excess SnCl_2_. GA was found capable to form a complex with SnCl_2_ that is evenly distributed on perovskite grains. The characteristics of the GA can be derived from the aromatic ring’s hydroxyl groups (–OH), which can donate electrons and absorb oxygen by hydrogen atoms. The SnCl_2_ layers present atop perovskite grains were expected to result in a wider bandgap compared to the bulk. After 1000 h of air exposure, unencapsulated GA-based devices preserved more than 80% of their initial PCE, which is one of the highest reports. Moreover, the solar cells with GA exhibited a high PCE of 9.03%.

Ascorbic acid (AA). It is a simple but effective additive that inhibits the oxidation of Sn^+2^ also regulates its film crystallization and creation, and can be utilized to build polymer-stabilized Pb/Sn binary PSCs [113], enhancing the optoelectronic quality of dual, perovskite films greatly. The resulting MA_0.5_FA_0.5_Pb_0.5_Sn_0.5_I_3_ film’s photogenerated carrier lifetime (183 ns) demonstrates this. As a result, MA_0.5_FA_0.5_Pb_0.5_Sn_0.5_I_3_ treated with AA achieved a high PCE of 14.01% and a higher stability than the control device employing the SnF_2_ additive, outperforming it. This research proposes a novel method for improving the performance and obtaining more stable Pb/Sn-PSCs.

### 5.2. Surface Modifiers

There are many surface modifiers for TPSCs that are applied using different methods and can be applied before or after the perovskite layer deposition. Controlling the surface terminations can majorly affect the stability and morphology [59,114,115].

One of the common examples is the introduction of antioxidant-carrying 4-fluorobenzohydrazide (FBH) on top of FASnI_3_ perovskite films [116]. The C=O group in such modifier was observed to interact with Sn^+2^ and promote the formation of largely oriented perovskites. Additionally, it was found that FBH results in the reduction in Sn^+4^ by the hydrazide group. According to the performed density functional theory calculation, the oxygen absorption barrier is increased after the FBH modification, resulting in a delay in the oxidation process. As a result of such, the interface modifier (capping layer) in the PCE increased from 8.34% to 9.47%.

Similarly, a dense layer of Al_2_O_3_ as a buffer layer separating perovskites and HTL can prevent degradation from moisture [117]. Cetyltrimethylammoniu bromide (CTAB) doped zirconium oxide (ZrO_x_) can also act in a similar manner [118]. In general, the switch from Pb to Sn affects the morphology severely due to the higher Lewis acidity of Sn^+2^ compared to Pb^+2^ [50]. Therefore, one of the major goals when making Sn films is to achieve compact and pinhole-free thin films.

### 5.3. Cation Engineering

Cations play a major role in Sn halide perovskites thin films in regard to lattice strain engineering. Nishimura et al. [119] investigated the relationship between lattice strain in tin-based perovskite films and TPSCs efficiency. They prepared tin-based Q*_x_*(FA_0.75_MA_0.25_)_1−*x*_SnI_3_ perovskites, where Q is various cations with different ionic radii such as Na^+^, K^+^, Cs^+^, BA^+^, and ethylammonium (EA^+^). The link between actual measured lattice strain and solar performance was explored. As the tolerance factor approached unity, the lattice strain decreased (measured by the Williamson hall plot of XRD data). As the lattice strain decreased, the performance of the Sn perovskites was enhanced. EA_0.1_(FA_0.75_MA_0.25_)_0.9_SnI_3_ with the lowest lattice strain yielded the best performance, because carrier mobility increased as lattice strain decreased. These lattice strains would disrupt carrier mobility and reduce solar cell performance [119]. The lowest lattice strains were found for Cs-0.1 and EA-0.1 and provided the highest mobility of about 43 cm^2^ V^−1^·s^−1^; however, in the case of Na-0.1, the lattice strain was found to be higher and therefore the mobility was down to 4.6 cm^2^ V^−1^·s^−1^.

Sun et al. [120] added a bi-linkable reductive cation (i.e., formamide (FM)), into FASnI_3_ to function as molecular glue for improving the stability and performance of TPSCs by the formyl group (–CHO) and amine group (−NH_2_). They revealed that the NH_2_ and C=O groups in FM are capable of interacting with FA^+^ and Sn^+2^ through hydrogen bonds and Lewis acid–base coordination, respectively. This resulted in a greater grain size, preferred orientation, lower defect density, and better film stability. The TPSC device based on 10% of FMI resulted in a 40% increased PCE from 5.51% to 7.71% with notable enhanced stability, retaining its initial PCE after one year in N_2_ without encapsulation. 

Jokar et al. [17] used guanidinium cations (GA^+^) as an additive with at least 1% of ethylenediammonium diiodide (EDAI_2_) to form a FASnI_3_ films, and this resulted in the remarkably improved performance of TPSCs. A high PCE of 8.5% was achieved and increased to 9.6% after 2000 h of storage in a glove box. Additionally, the resulting perovskite operated for almost an hour under continuous illumination and for six days in air without encapsulation [17].

### 5.4. Solvent Engineering 

Solvents and secondary solvents can play a major role in the formation of pinholes in thin films, especially thin films that contain organic materials [121]. One common way to produce a compact Sn film is by different solvents, such as dimethylformamide (DMF) or dimethyl sulfoxide (DMSO) [122,123]. Post-treatment with antisolvents also plays a crucial role in the quality of tin-based perovskite films [123,124].

It was reported that when perovskite films are formed without any anti-solvent dripping, the precursor is far from supersaturated until the solvent totally evaporates and, as a result, the nucleation site density is rather low, resulting in a flower-like film with limited surface coverage [123]. Therefore, different antisolvents (diethyl ether (DE), toluene (TL), and chlorobenzene (ClB)) were tested. The size of pinholes in the FA_0.75_MA_0.25_SnI_3_ films dripped by DE is less than that of pinholes in the film dripped by TL, but the quantity of pinholes is greater. In the case of ClB, the film has a uniform surface with full coverage and clear grain features. One reason for this could be related to its high boiling point of 131 °C, which has a slower evaporation rate, which extends the crystal development period during the thermal annealing process.

Hao et al. [42] investigated the DMSO function as a the Lewis base solvent to adjust the crystallization rate of MASnI_3_ perovskite by Lewis acid–base interaction. When DMSO molecules react with Lewis acid SnI_2_, they form the SnI_2_-3DMSO intermediate adduct, which effectively slows down the interaction between MAI and SnI_2_, resulting in an enhanced MASnI_3_ film. Similarly, the generation and orientation of FASnI_3_ perovskites was controlled when poly-ethylene-co-vinyl acetate (PEVA) was introduced into the anti-solvent [125]. At the grain boundaries, the C=O groups of PEVA molecules cause a complexation between Lewis acid–base interaction and Sn^+2^. This results in larger grains and lowered surface defects of FASnI_3_, leading to enhanced devices’ performance.

### 5.5. Low-Dimensional Perovskites

In fact, using different solvents incorporated with different additives as well as cations with different radii often has a direct influence on the dimensionality of the perovskite films, which is on its own is a major research and development direction. Lower dimension perovskites seem to be more stable than three-dimensional ones, so these are expected to improve tin-based perovskites’ stability [126]. The three-dimensional structure of perovskites could be decreased to two- or one-dimensional by substituting the bulky organic ammonium ions at the A-site in the perovskite lattice or by inserting 2D materials in the precursor solution [127,128]. 2D perovskites seems to have exceptional optoelectronic properties and therefore may make them excellent photovoltaic materials [129]. 2D perovskite reduces moisture as well as oxygen from going inside the film [130,131]. It also can reduce defects, resulting in a low amount unwanted self-doping [132]. Many researchers are currently investigating 2D TPSCs to increase their stability based on the benefits of lower dimension perovskites.

Similarly, low-dimensional Sn perovskites have become a topic of interest in TPSCs due to their ability to improve device performance and stability. In 2017, Liao et al. [133] incorporated phenylethylammonium (PEA) into FASnI_3_ perovskites and they achieved perpendicularly oriented, low-dimensional Sn perovskite films with remarkably enhanced stability and a PCE of 5.9%. In 2020, a report by Liang et al. [134] utilizing indene-C_60_ bisadduct (ICBA) as an ETM found that their Sn-based perovskite (PEA_x_ FA_1−x_SnI_3_) with PEA incorporation formed a low-dimensional perovskite with reduced defect concentrations, which resulted in a high *V_OC_* of 0.94, a record PCE of 12.4%, and better stability (shelf stability of 3800 h).

### 5.6. Variety of Very Recent Perovskite Additives, Surface/interface Modifiers in TPSCs with Noticeable Performance

There are many additives that were reported in recent years to enhance the performance of TPSCs [55,72,135,136,137]. However, here (Table 1) we summarize most of the very recent and noticeable additives and correlated outstanding device performance of lead-free TPSCs that were reported during the last two years. Table 2 provides a list of the most recent and highly performing surface/interface modifiers that were applied in lead-free TPSCs. Table 3 provides a list of most recent highly performing mixed Pb-Sn perovskites with Pb ≤ 50% and it includes devices with both perovskite additives and surface/interface modifiers. The table also provides structure and reported stability.

It is noteworthy to mention that the reasons behind the enhancement of the performance of the devices listed in the tables are often related to the same reasons. Here, we briefly list the reasons behind the enhancement in general, which is important for further future consideration and development to achieve even higher performance: (i) oxidation, (ii) reduced defects, (iii) controlled crystallization, (iv) morphology (compactness and pinholes, strain relaxation), (v) charge diffusion and extraction (mobility, carriers density, energy levels, recombination), (vi) built-in electric field (gradient vertical perovskite growth), (vii) better choice of cations (reduced or eliminated MA^+^) (iix) hydrophobicity, and (ix) passivation of the acidic and hygroscopic surface of the commonly used PEDOT:PSS HTL (or alternative or HTL or SAMs).

## 6. Conclusions and Prospects

Currently, there is a significant amount of research that is being carried out on the enhancement of TPSCs. The PCE of lead-free as well as lead-mixed TPSCs is approaching 15% and 24%, respectively. Similarly, the stability of TPSCs is rapidly improving. It may be true that the PCEs and the stability of TPSCs are still behind lead PSCs; however, TPSCs have a wider range of applications, especially due to the lesser toxicity and narrower bandgap of Sn perovskites.

The development of new and improved synthetic methods for Sn-based perovskite materials can help to further boost the efficiency, stability, and scalability of TPSCs. Further investigation of new device structures can lead to further improvements in efficiency and stability. Investigating the use of new interfaces and interlayers can help to improve the stability and efficiency of Sn-based perovskite solar cells. Improving the stability of Sn-based perovskite solar cells is still a crucial direction for future research. Strategies such as passivation of defects, encapsulation, and material engineering can help to improve their long-term stability.

It is important to emphasize that Sn-based perovskites exhibit both metallic and semiconducting behavior depending on the preparation method. This is attributed to the presence of metallic Sn phases in the perovskite film at different levels. Therefore, different properties can be observed across the film (bottom surface, bulk, and top surface). This behavior can result in a built-in electric field. In general, this varying conducting nature may result in a trade-off between electrical conductivity and photoelectric performance. As discussed, various strategies have been proposed to reduce the metallic Sn content, and recently, there have been several good attempts to control such problem and to use it to make a controlled built-in field and surfaces. In general, there are several challenges facing TPSCs and manifested in the poor stability of Sn. Additionally, the control of crystallization dynamics (growth and gradient), self-doping, morphology, strains, defects density, oxidation, ion migration, and charge transport and extraction all hinder the performance of TPSCs. However, many successful efforts have been made to resolve these issues, such as the use of various additives, surface/interface modifiers, solvents engineering, crystal dimensionality, and compositional engineering, which are all moving forward in the enhancement of the overall PV performance and stability of TPSCs.

This review summarizes some milestones in the development and the up-to-date progress of tin perovskites and TPSCs. The crystal structure and electrical properties of tin perovskite materials, as well as the cause of their chemical instability, were discussed. Furthermore, the main factors affecting the stability and resulting degradation, such as moisture, oxygen, ion migration, and UV-illumination, were summarized.

## Figures and Tables

**Table 1:** Best performing Pb-free TPSCs with perovskite additives reported in 2021 and 2022.

Structure	Additive	PCE (%)	Eg (eV)	Stability (Period, Conditions, Percentage from Original Efficiency)	
ITO/PEDOT ^a^/FA_0.92_PEA_0.08_SnI_3_/PCBM/Al	MACl	7.1	1.42	42 days, encapsulated, 100+% 6 h, air, 60%	[138]
FTO/c-TiO_2_/mp-TiO_2_/CsSnI_3_/P3HT/Au *	MBAA ^b^	7.5	1.3	60 days, nitrogen, 60% 5 days, air, 76.5% 5 days, 1 sun, 58.4%	[139]
ITO/PEDOT/FASnI_3_/C60/BCP/Ag **	FM^+ t^	7.7%	1.4	367 days, nitrogen, 100%	[120]
FTO/SnO_2_/Al_2_O_3_-Gr ^c^/FA_0.8_MA_0.2_SnI_3_/spiro ^d^/Au *	rGO ^e^	7.7	1.27	30 days air, 42% 30 days, 85%, dry argon	[140]
ITO/PEDOT/FA_0.75_MA_0.10_SnI_2_Br/PCBM/BCP/Ag	PEA^+ f^	8.0	1.66	63 days, nitrogen, 100% 13 days, air, 100%	[141]
ITO/PEDOT//PCBM/PCB/Ag	F-PDI ^s^	9.5	1.42	125 days, 1 sun, nitrogen, 80%	[142]
ITO/PEDOT/MASnI_3_/PCBM/BCP/Ag	EABr ^g^	9.6	1.3	30 days, nitrogen, 93%	[143]
FTO/PEDOT/FASnI_3_/C_60_/BCP/Ag	FAAc ^h^	10.0	NA	67 days, 1 sun, nitrogen, 82%	[144]
PET/ITO/NiO_x_/FASnI_3_/4AMPI2 ^i^/PCBM/BCP/Ag ***	Ge/GeO_2_	10.4	1.38	29 days, 1 sun, nitrogen, MPP, 80% 2500 bending cycles, R = 5 mm, 80%	[145]
ITO/PEDOT/FASnI_3_/C_60_/BCP/Ag **	DipI ^j^ and NaBH4 ^k^	10.6	1.38	54 days, nitrogen, MPP, 96%	[146]
ITO/PEDOT/FASnI_3_/PCBM/BCP/Ag	EABr ^g^	10.8	1.48	84 days, nitrogen, 82%	[147]
ITO/PEDOT/FASnI_3_/PAI ^l^/C_60_/BCP/Ag	PEA^+ f^	12.1	1.4	21 days, 1 sun, MPP, encapsulated, 94%	[148]
ITO/PEDOT/MASnI_3_/ICBA/BCP/Ag	CsPbI_3_ QDs ^m^	12.5	1.3	40 days, nitrogen, 96% 23 days, 1 sun, 62%	[149]
ITO/PEDOT/FASnI_3_/ICBA/BCP/Ag	CsPbI_3_ QDs	13.0	1.4	40 days, nitrogen, 83% 23 days 1 sun, 64%	[149]
ITO/PEDOT/FASnI_3_+PHCl-Br/C_60_/BCP/Ag	PhNHNH_3_^+^ and Ph-Cl^−^ Br^− n^	13.4	1.4	14 days, 1 sun, 82% 200 days, nitrogen, 91%.	[69]
ITO/PEDOT/FASnI_3_/ICBA/BCP/Ag	4A3HA ^o^	13.4	1.4	83 days, nitrogen, 98% 42 days, at 82 °C, 80%	[150]
ITO/PEDOT/PEAFASn(IBr)_3_/ICBA/BCP/Ag	GAA ^p^	13.7	NA	50 days, nitrogen, 93%	[151]
ITO/PEDOT/FASnI_3_/BCP-ICBA/Ag	3T ^q^	14.0	1.4	30 days, nitrogen, 100% 9 h, air, 85%	[152]
ITO/PEDOT/FASnI_3_/ICBA/BCP/Ag ****	PEA ^f^ Br	14.6	NA	100 days, nitrogen, 96%	[153]
ITO/PEDOT/FASnI_3_/ICBA/BCP/Al	FPEABr ^r^	14.8	1.43	19 days, nitrogen, 80%	[154]

^a^ PEDOT:PSS (PEDOT), ^b^ N,N′-methylenebis(acrylamide), ^c^ Graphene (Gr), ^d^ spiro-OMeTAD, ^e^ reduced graphene oxide (rGO), ^f^ phenylethylammonium (PEA^+^), ^g^ ethylammonium bromide (EABr), ^h^ formamidine acetate (FAAc), ^i^ 4-(aminomethyl) piperidinium diiodide (4AMPI2), ^j^ Dipropylammonium iodide (DipI), ^k^ sodium borohydride (NaBH4), ^l^ n-propylammonium iodide (PAI), ^m^ quantum dots(QDs), ^n^ phenylhydrazine (PhNHNH^3+^) and phenylhalides (Cl^−^ and Br^−^) (Ph-Cl^−^Br^−^), ^o^ 4-amino-3-hydroxybenzoic acid (4A3HA), ^p^ 2-Guanidinoacetic acid (GAA), ^q^ trimethylthiourea (3T), ^r^ 4-fluoro-phenethylammonium bromide (FPEABr), ^s^ fluorinated-perylene diimide (F-PDI), ^t^ formamide (FM), * n-i-p structure, ** one of the best stability records, *** flexible record and with NiO_x_ HTL, **** with SnI_2_-DMSO colloidal complex.

**Table 2:** Surface and Pb-free. Best-performing Pb-free TPSCs with perovskite surface/interface modifiers reported in 2021 and 2022.

Structure	Treatment	PCE (%)	Eg (eV)	Stability (Period, Conditions, Percentage from Original Efficiency)	
ITO/PEDOT ^a^/CsSnI_3_/C_60_/BCP/Cu	TSC ^b^ on SnI_2_	8.2	N.A.	21 days, 1 sun, encapsulated, 71%	[155]
ITO/PTAA/FASnI_3_/C_60_/BCP/Ag	PEAI ^c^ on PTAA	8.3	1.4	83 days, nitrogen, 87%	[56]
FTO/PEDOT/EDA_0.01_(GA_0.06_(FA_0.8_Cs_0.2_)_0.94_)_0.98_SnI_2_Br/C_60_/BCP/Ag	2PACz^d^ on PEDOT	8.7	1.62	70 days, nitrogen, 75%	[156]
FTO/bl-TiO_2_/mp-TiO_2_/Cs_0.1_FA_0.9_SnI_3_/PTAA/Au *	ThMAI ^e^ on PVSK	9.1	1.45	35 days, nitrogen, 92%, 6 days, air, 62%	[157]
ITO/NiO_x_/FASnI_3_/PCBM/BCP/Ag	FAAc ^f^ on PVSK	9.1	N.A.	55 days, nitrogen, 80%	[158]
ITO/PEDOT/FA_0.75_MA_0.25_SnI_2.75_Br_0.25_/PCBM/BCP/Ag	CF_3_PEAI ^g^ on pvsk	10.4	1.45	52 days, nitrogen, 80%, 4 days, air, 80%	[159]
ITO/PEDOT/FA_0.98_EDA_0.01_SnI_3_/C_60_/Ag	SA ^h^ + PEDOT on PEDOT	10.5	1.33	83 days, nitrogen, 95%	[160]
FTO/PEDOT/FA_0.75_MA_0.25_SnBrI_2_/ICBA/Bphene ^i^/Ag **	KSCN ^j^ on PEDOT	11.2	1.63	42 days, nitrogen, 80%	[161]
FTO/PEDOT/FASnI_3_/C_60_/BCP/Ag	vapor of EDA ^k^ on PVSK.	11.3	1.42	40 days, nitrogen, 85%	[162]
ITO/PEDOT/FASnI_3_/C_60_/BCP/Ag ***	PAI ^l^ on PVSK	12.1	1.4	21 days, 1 sun, MPP, encapsulated, 94%	[148]
ITO/PEDOT/FASnI_3_/C_60_/BCP/Ag	PMMA ^m^ on PVSK	13.8	1.41	42 days, 1 sun, encapsulated, MPP, 94%	[163]
ITO/PEDOT/FAMASnI_3_/C_60_/BCP/Ag	FACl on PVSK	14.7	1.42	42 days, nitrogen, 92%	[164]

^a^ PEDOT:PSS (PEDOT), ^b^ thiosemicarbazide (TSC), ^c^ phenylethylammonium iodide (PEAI), ^d^ carbazole with phosphonic acid (2PACz), ^e^ 2-thiophenemethylammonium iodide (ThMAI), ^f^ formamidine acetate (FAAc), ^g^ 3-(trifluoromethyl) phenethylamine hydroiodide (CF_3_PEAI), ^h^ zwitterion, sulfamic acid (SA), ^i^ bathophenanthroline (Bphene), ^j^ potassium thiocyanate (KSCN), ^k^ ethane-1,2-diamine (EDA), ^l^ n-propylammonium iodide (PAI), ^m^ poly-methyl methacrylate (PMMA). * n-i-p structure, ** Indoor PCE-record of 17.6% under 1062 lx, *** cold precursor solution (0 °C).

**Table 3:** Additives/surface and Pb ≤ 50%. Best-performing TPSCs with lead content ≤ 50% with perovskite surface/interface modifiers or perovskites additive reported in 2021 and 2022.

Structure	Additive/Treatment	PCE (%)	Eg (eV)	Stability (Period, Conditions, Percentage from Original Efficiency)	
ITO/PEDOT ^a^/FASn_0.5_Pb_0.5_I_3_/C_60_/BCP/Ag	K-SCN ^b^ additive	14.5	1.25	5 days, air, 55%	[165]
ITO/PEDOT/FA_0.8_MA_0.15_Cs_0.05_Pb_0.5_Sn_0.5_I_3_/C_60_/BCP/Ag	PEAI ^c^ additive	17.3	1.25	33 h, air, 85% 45 days, nitrogen, 87%	[166]
ITO/FA_0.85_Cs_0.15_Sn_0.5_Pb_0.5_I_3_/PCBM/PCB/Cu *	FSA ^d^ additive and PEAI ^c^ in toluene on PVSK	17.4	1.27	20 days, air, 81%	[167]
ITO/PEDOT/FA_0. 5_MA_0.5_Pb_0.5_Sn_0.5_I_3_/PCBM/C_60_/BCP/Ag	IMBF4 ^e^ additive	19.1	1.25	42 days, nitrogen, 90% 2 days, 1 sun, 80%	[168]
ITO/PEDOT/FA_0.83_Cs_0.17_Pb_0.5_Sn_0.5_I_3_/C_60_/BCP/Ag	PEAI ^c^ on PVSK	19.1	NA	4 days, nitrogen, 1 sun, MPP 82%	[169]
ITO/NiO_x_/FA_0.5_MA_0.5_Sn_0.5_Pb_0.5_I_3_/PC_61_BM/BCP/Ag	PFN ^f^ on NiO_x_	19.8	1.26	20 days, air, 68%	[170]
ITO/PEDOT/FA_0.7_MA_0.3_Pb_0.5_Sn_0.5_I_3_/PCBM/BCP/Cu.	CA ^g^ additive	19.9	1.26	21 days, nitrogen, 90%	[171]
ITO/Cs_0.05_MA_0.45_FA_0.5_Pb_0.5_Sn_0.5_I_3_/PCBM/C_60_/BCP/Ag *	Cu-SCN ^b^ and GlyHCl ^h^ on ITO	20.1	1.21	42 days, nitrogen, 90% 4 days, 1 sun, MPP, 72%	[172]
ITO/PEDOT/FA_0.7_MA_0.3_Pb_0.5_Sn_0.5_I_3_/PCBM/BCP/Ag	[PNA]BF4 ^i^ on PEDOT	20.1	NA	10 days, nitrogen, 85 °C, 80% 50 days, nitrogen, 90.8%	[173]
ITO/PEDOT/FA_0.7_MA_0.3_Pb_0.5_Sn_0.5_I_3_/C_60_/BCP/Ag	PhDMADI ^j^ additive	20.5	1.25	29 days, nitrogen, 95%	[174]
ITO/PEDOT/MA_0.3_FA_0.7_Pb_0.5_Sn_0.5_I_3_/PCBM/BCP/Ag	GUA ^k^ additive and HAI ^l^ on PVSK	20.5	1.27	6 days nitrogen, 1 sun, 60%	[175]
FTO/PEDOT/Cs_0.025_FA_0.475_MA_0.5_Sn_0.5_Pb_0.5_I_2.925_Br_0.075_/PCBM/C_60_/BCP/Ag	RbI additive	21.0	1.28	6 days, nitrogen, at 85 °C, 75% 30 days, nitrogen, 99%	[176]
ITO/PEDOT/FA_0.5_MA_0.5_Pb_0.5_Sn_0.5_I_3_/C_60_/BCP/Ag	HZBA ^m^ additive	21.1	1.26	8 days, nitrogen, 90%	[177]
ITO/PEDOT/Cs_0.2_FA_0.8_Pb_0.5_Sn_0.5_I_3_/C_60_/BCP/Cu	BaI_2_ additive	21.2	1.21	15 days, encapsulated, 1 sun, MPP, 95%	[178]
FTO/PEDOT/FA_0.6_MA_0.4_Sn_0.6_Pb_0.4_I_3_/C_60_/BCP/Ag	N,Cl-GQDs ^o^ at PEDOT	21.5	1.25	42 days, nitrogen, 90%	[179]
ITO/PEDOT/Cs_0.05_FA_0.7_MA_0.25_Sn_0.5_Pb_0.5_I_3_/C_60_/BCP/Ag	BBMS ^n^ + SnF_2_	22.0	1.22	111 days, nitrogen, 60 °C, 98 %	[180]
ITO/PEDOT/FA_0.6_MA_0.4_Sn_0.6_Pb_0.4_I_3_/C_60_/BCP/Ag.	PEAI ^c^ and guanidinium-SCN ^b^	22.1	1.25	76 days, nitrogen, MPP, 82%	[181]
ITO/CzAn^p^/PMMA/FA_0.8_Cs_0.2_Sn_0.5_Pb_0.5_I_3_/PCBM/C_60_/BCP/Cu	CzAn ^p^ HTM and BHC ^q^ on PVSK	22.6	1.22	7 days, encapsulated, MPP, 1 sun, 90% 42 days, encapsulated, 96%	[182]
FTO/Cs_0.025_FA_0.475_MA_0.5_Sn_0.5_Pb_0.5_I_2.925_Br_0.075_/EDA ^r^/PCBM/C_60_/BCP/Ag *	2PACz ^s^ and MPA ^t^ at FTO	23.3	1.25	42 days, nitrogen, 1 sun, 100%	[183]
FTO/PEDOT/Cs_0.1_FA_0.6_MA_0.3_Sn_0.5_Pb_0.5_I_3_/C_60_/BCP/Ag	EDAI_2_^u^ on PVSK and GlyHCl ^v^ at PEDOT	23.6	1.24	8 days, nitrogen, 1 sun, MPP, 80%	[184]

^a^ PEDOT:PSS (PEDOT), ^b^ thiocyanate (SCN), ^c^ 2-phenylethylazanium iodide (PEAI), ^d^ formamidine sulfinic acid (FSA) additive, ^e^ ionic imidazolium tetrafluoroborate (IMBF4), ^f^ poly[(9,9-bis(30-(N,N-dimethylamino)propyl)-2,7-fluorene)-alt-2,7-(9,9-dioctyfluorene)] (PFN), ^g^ caffeic acid (CA), ^h^ glycine hydrochloride (GlyHCl), ^i^ iso-pentylammonium tetrafluoroborate salt ([PNA]BF4), ^j^ p-phenyl dimethylammonium iodide (PhDMADI), ^k^ β-guanidinopropionic acid (GUA), ^l^ hydrazinium iodide (HAI), ^m^ 4-hydrazinobenzoic acid (HZBA), ^n^ 1-bromo-4-(methylsulfinyl) benzene (BBMS), ^o^ graphene quantum dots (GQDs), ^p^ poly[(phenyl)imino[9-(2-ethylhexyl)carbazole]-2,7-diyl] (CzAn), ^q^ benzylhydrazine hydrochloride (BHC), ^r^ Ethylenediamine, ^s^ 2-(9H-carbazol-9-yl) ethyl] phosphonic acid (2PACz), ^t^ methyl phosphonic acid (MPA), ^u^ ethylenediammonium diiodide (EDAI_2_), ^v^ glycine hydrochloride (GlyHCl), * HTL-free.
